# Iatrogenic injury of the popliteal artery in orthopedic knee surgery: clinical results and development of a therapeutic algorithm

**DOI:** 10.1007/s00068-022-01961-8

**Published:** 2022-03-31

**Authors:** Yvonne Gosslau, Tobias Dominik Warm, Stefan Foerch, Sebastian Zerwes, Christian Scheurig-Muenkler, Alexander Hyhlik-Duerr

**Affiliations:** 1 Vascular Surgery, Faculty of Medicine, University of Augsburg, Stenglinstr. 2, 86156 Augsburg, Germany; 2 Trauma Surgery, Faculty of Medicine, University of Augsburg, Stenglinstr. 2, 86156 Augsburg, Germany; 3 Diagnostic and interventional Neuroradiology, Faculty of Medicine, University of Augsburg, Stenglinstr. 2, 86156 Augsburg, Germany

**Keywords:** Popliteal artery injury, Vascular injury, Iatrogenic injury, Knee surgery, Therapeutic algorithm

## Abstract

**Purpose:**

Intraoperative injury to the popliteal artery is a rare complication of orthopedic surgery, however, it can have serious consequences, including major amputation. Recommendations for a standard approach are lacking. The aim of this study was to develop an interdisciplinary therapeutic algorithm to assist in complication management.

**Methods:**

From 01/11 to 12/20, 16 arterial injuries after knee surgery were analyzed in a retrospective single-center study. Four cases involved recurrent orthopedic surgery. Procedures performed included eleven total knee arthoplasties (TKA), two TKA replacements, one arthroscopy, and two high tibial osteotomies. Clinical presentation of patients was hemorrhage (*n* = 2), ischemia (*n* = 7), the combination of both (*n* = 4), or pseudoaneurysm formation (*n* = 3).

**Results:**

Ten patients underwent endovascular treatment, some as combined procedures: (stent)-PTA (*n* = 6), aspiration thrombectomy (*n* = 5), thrombin injection (*n* = 1), and embolization (*n* = 1). Six patients were treated surgically: four with bypass/interposition and one with a patch plasty and one as a hybrid procedure, respectively. Only autologous great saphenous vein was used. All extremities could be preserved. Functional impairment remained in six cases.

**Conclusion:**

Both endovascular and surgical procedures can be used to treat arterial injuries after knee surgery. Efficient standardized diagnosis and the involvement of vascular expertise are essential to prevent functional impairment or limb loss, as suggested in the algorithms.

## Introduction

The knee joint, as the largest load-bearing joint in the body, is frequently affected by arthritic pathologies. In Germany, for example, up to 90% of people over the age of 60 suffer from gonarthrosis. After conservative treatment options have been exhausted, modern orthopedic surgery offers various surgical solutions. For joint preservation, perigenicular osteotomies have been a proven therapeutic procedure for decades. Most commonly, a high tibial osteotomy (HTO) is performed.

If joint preservation is not reasonably possible, the procedure of choice is total knee arthroplasty (TKA). This is one of the most frequent surgical procedures in Germany with about 180 000 cases/year [1].

Surgical therapy aims at improving the knee joint function and quality of life for the patient.

Considering this, complication rates of about 10% for osteotomies and 1.4% for TKA primary procedures or 3.24% for revision surgery are not negligible [1]. The incidence of vascular injury in these cases is estimated to be 0.01–0.51% [2–4]. Due to the possibility of disastrous consequences for the patient up to major amputation, an injury of the popliteal artery is a rare but particularly feared event, which can frequently result in protracted litigation [5].

Regarding this rare complication, the previous research reports cover mainly single cases and publications from national registries. Recommendations for complication management are completely lacking.

The present study reports results of the largest case series of arterial injuries in the course of orthopedic knee joint surgery. Our interdisciplinary experience in the management of this complication and the results of this investigation have led to the development of the algorithms presented here. They are intended to be a structured tool for the treating physicians in case of complication to reduce the risk of long-term impairment and to improve the outcome for the patients.

## Patients and methods

In the period of January 2010–December 2020, 16 patients with iatrogenic popliteal injury which occurred during orthopedic knee surgery were treated at Augsburg University Hospital.

In our single-center analysis, data since 2016 were extracted from a prospectively maintained vascular trauma registry. Prior years were obtained retrospectively from electronic patient files. All documented iatrogenic trauma-induced popliteal injuries were included. All study participants were transferred to our department from orthopedic hospitals without a vascular surgeon.

Data on demographics were collected as well as initial treatment diagnosis. General data on orthopedic and vascular surgeries or interventions, such as ischemia time, duration of therapy, blood loss, and need for fasciotomy, were also recorded. Outcome was documented using the following parameters: need for amputation, functional integrity, and long-term consequences, such as edema or chronic pain.

Patients were actively invited to a follow-up examination. It included a standardized examination procedure with ultrasound control, hemodynamic measurements, and a questionnaire regarding functional impairment.

Data acquisition was performed using Microsoft Excel^®^ (version 16.43; Microsoft Corporation, Redmond, WA, USA). SPSS (version 27.0; IBM Corp., Armonk, NY, USA) provided the Fisher's exact test to evaluate significance.

The local Ethics Board of Augsburg University Hospital granted approval for the investigation (No. 2020-37).

## Results

Patients were heterogeneous with respect to age and previous diseases; no significant differences were found between surgical and interventional treatment. Demographic data are shown in Table 1.

**Table 1:** Patient characteristics

	Total number *n* = 16	Endovascular therapy *n* = 10	Surgical treatment *n* = 6	*P* value
Sex (m/f/d)	10/6/0	5/5/0	4/2/0	0.63
Age (y)	67.50	72.00	53.50	0.09
Follow-up (d)	628.94 (2–2408)	615.50 (2–2408)	651.33 (19–2035)	0.88
ASA classification 1/2/3	3/7/6	1/5/4	2/2/2	0.52/0.63/1.00
Orthopedic disease				
Gonarthrosis Meniscal lesion Joint infection	13 1 2	8 1 1	5 0 1	1.00 1.00 1.00

*m*  male, *f* female, *d* divers, *y* years, *ASA* American Society of Anaesthesiologists

### Orthopedic surgery and clinical presentation of the complication

**Fig. 1:**
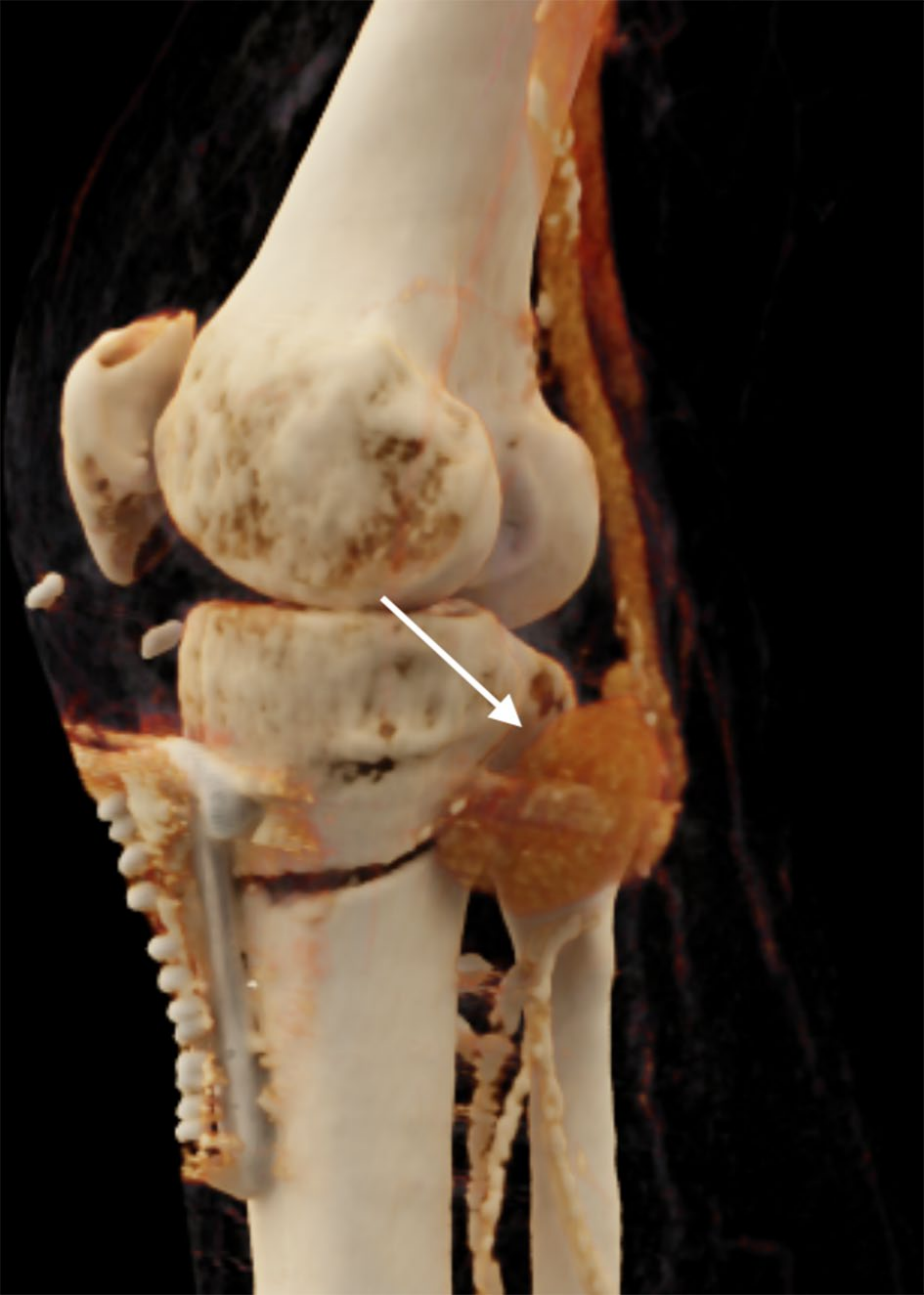
3D reconstruction of postoperative situs after high tibial realignment osteotomy from contrast-enhanced computed tomography. Shown is the course of the osteotomy and the spatial proximity to the popliteal artery, which shows contained rupture (arrow)

The range of procedures performed includes eleven TKAs, two TKA replacements, two HTOs (Fig. 1), and one genicular arthroscopy.

Four out of 16 operations were revision procedures: the two before mentioned TKA replacements, and one TKA implantation each after HTO or arthroscopy in patient history, respectively.

The duration of the orthopedic procedure was collected for eight procedures from the available reports and was 92 min on average, 89 min for initial procedures, and 113 min for revision procedures.

In 12 cases, a vascular injury was suspected either in the operating room (OR) or in the recovery room (PACU). In the remaining four cases, the diagnosis was made in the subacute interval, which was defined as ischemia duration longer than 24 h.

Intraoperative abnormalities were only documented in six surgical reports, ranging from descriptions of difficult access site, such as scarring, to the notion of increased bleeding.

Bleeding symptoms were documented in six cases, which were combined in four cases with signs of leg ischemia. Seven patients presented with isolated ischemia of the affected limb.

Three patients developed a pseudoaneurysm, which was symptomatic by compression of surrounding structures with swelling, sensorimotor deficit, and pain.

Twelve patients underwent orthopedic surgery with a tourniquet applied.

Only in one case, the intraoperative bleeding was so severe that transfer to the vascular surgery department was performed with tourniquet in place.

The other details on orthopedic surgery and clinical presentation are shown in Table 2.

**Table 2:** Orthopedic procedures and clinical presentation

	Total number *n* = 16	Primary surgery *n* = 12	Revision surgery *n* = 4	*p* value
TKA	11	9	2	0.55
TKA revision	2	0	2	0.05
Arthroscopy	1	1	0	1.00
HTO	2	2	0	1.00
Orthopedic surgery with tourniquet	12	9	3	1.00
Orthopedic surgery with anti-platelet therapy	1	1	0	1.00
Vascular anatomical variation	2	2	0	1.00
Abortion of the orthopedic procedure	1	1	0	1.00
Transfer with applied tourniquet	1	1	0	1.00
*Clinical presentation at the vascular unit*				
Bleeding	2	2	0	1.00
Ischemia	7	4	3	0.26
Bleeding and ischemia	4	3	1	1.00
Pseudoaneurysm	3	3	0	0.53

*TKA* total knee arthroplasty, *HTO* high tibial osteotomy

There was no information on preoperative status regarding CMS (circulation, motor function, sensibility) in the patient files.

Four patients suffered from peripheral artery disease (PAD) according to patient history.

### Diagnostic imaging

The imaging procedures performed following the suspicion of vascular injury were inconsistent.

In some cases, duplex sonography (CCDS) or computed tomography (CT) had already been performed in the orthopedic clinic.

After transfer to our vascular surgery department, an orienting CCDS was performed, followed by CT angiography (CTA) in 9 cases and digital subtraction angiography (DSA) in 13 cases.

**Fig. 2:**
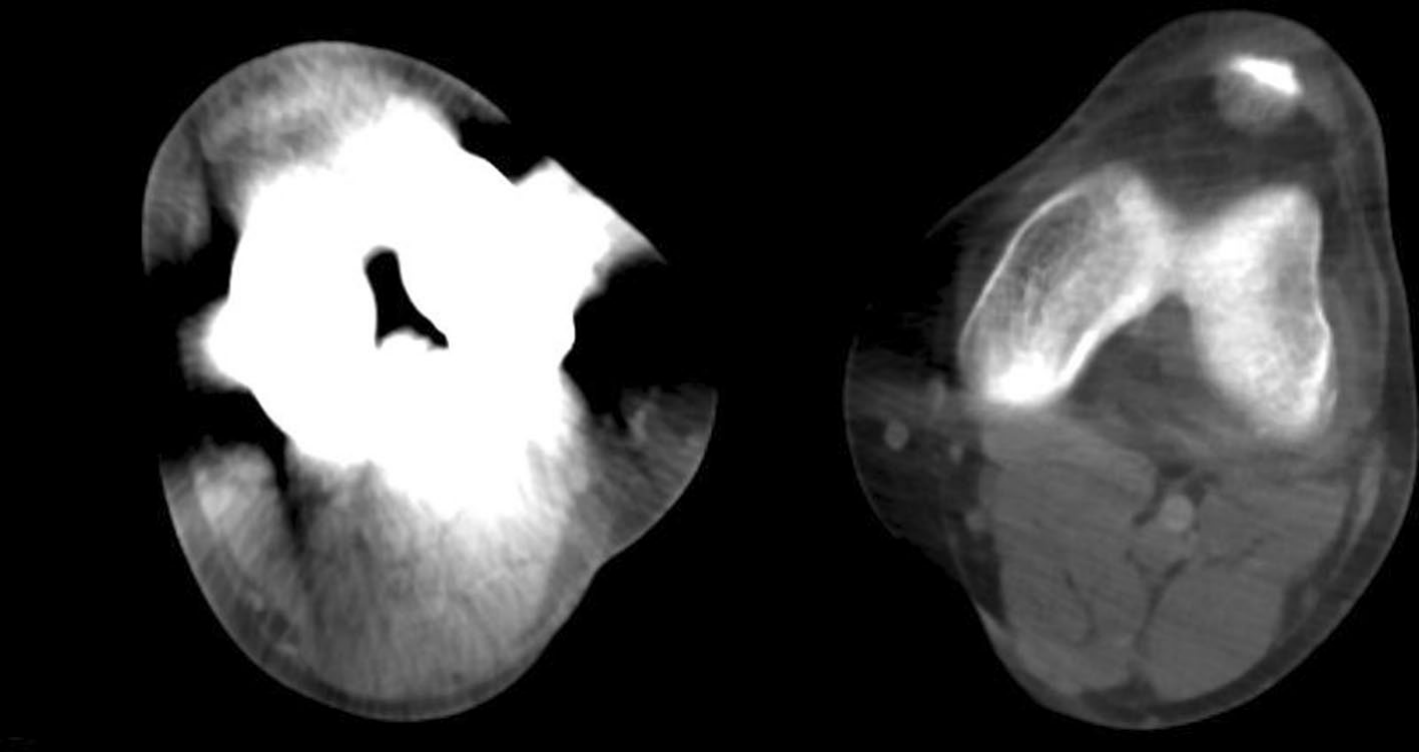
On postoperative computed tomography, assessment of the neurovascular structures near the knee joint gap is complicated by beam hardening artifacts around the metallic endoprosthesis

DSA was performed as a primary imaging modality as well as a supplementary tool after poorly assessable CTA to confirm the diagnosis (Fig. 2).

The popliteal artery was injured at the level of the knee joint gap in 14 cases.

In two cases, an anatomical variation with high branching of the anterior and posterior tibial arteries was present.

**Fig. 3:**
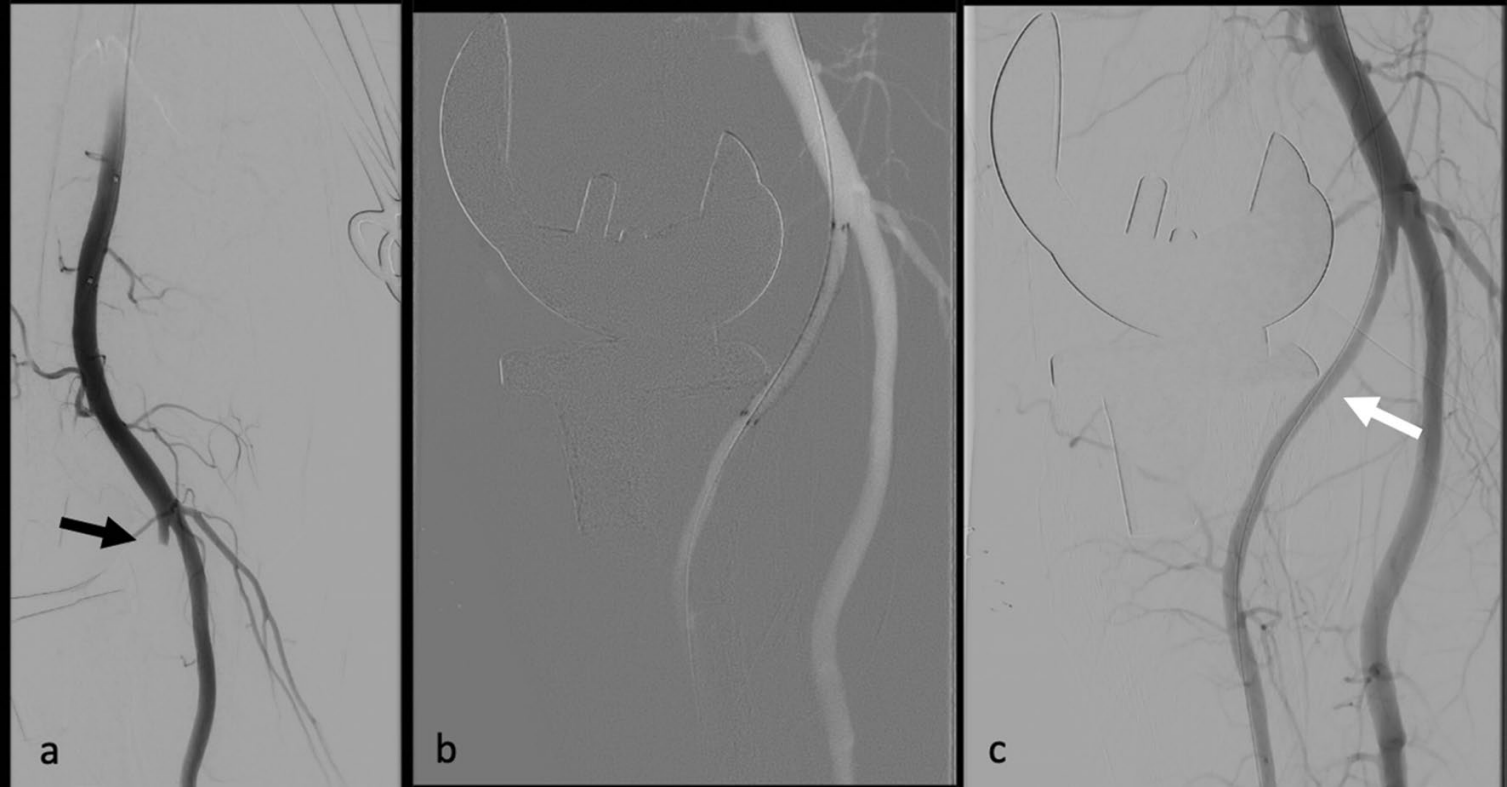
Angiographic image of a high branching anterior tibial artery. **a** The black arrow points to the abort of the vessel above the knee joint gap. **b** Recanalization of the vessel and insertion of a stent graft. **c** Postinterventional result; the white arrow illustrates the positional relationship between the vascular variability and the dorsal edge of the tibia

In these patients, it was always the anatomical variability affected by the injury (Fig. 3).

### Vascular treatment

**Fig. 4:**
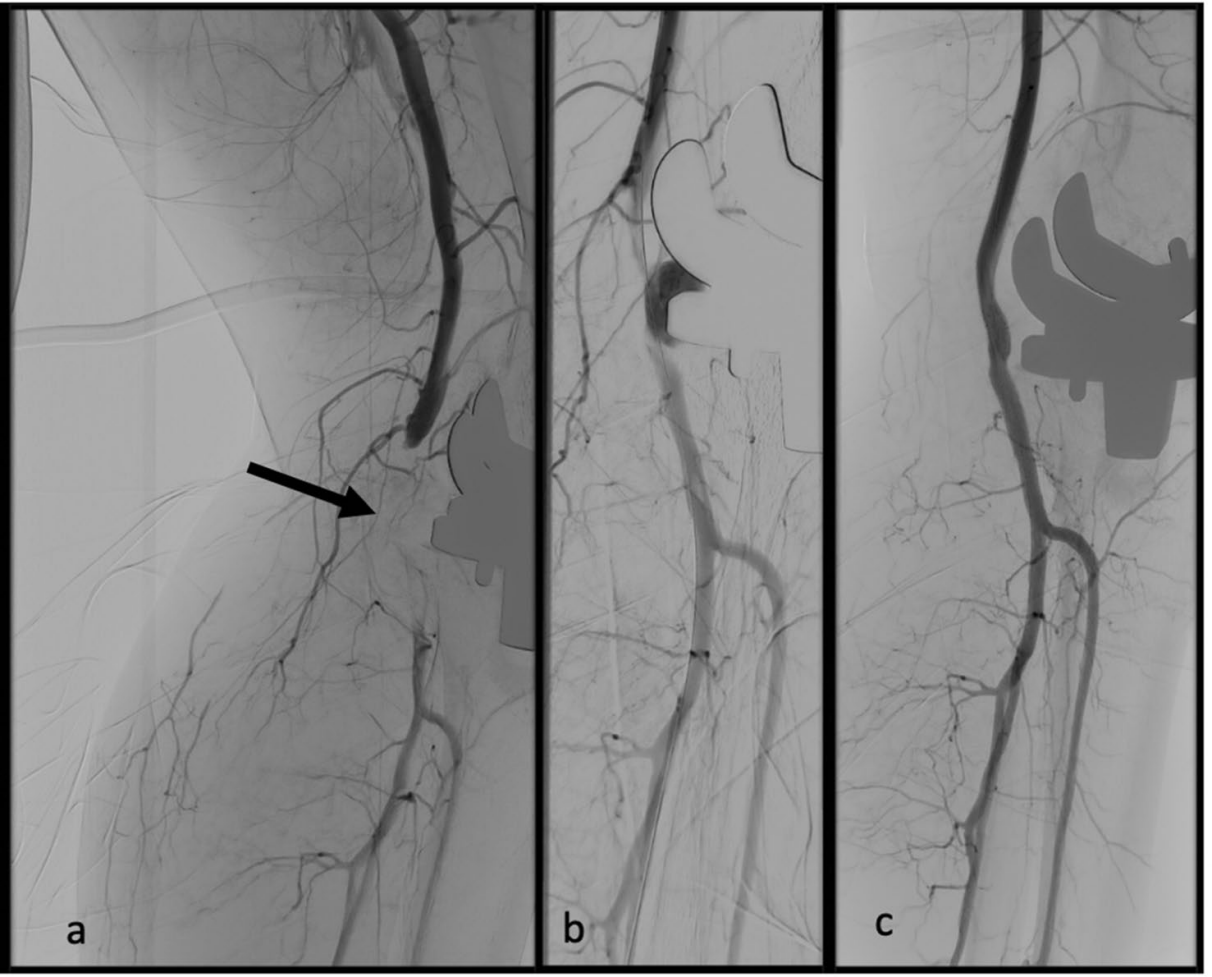
Occlusion of the popliteal artery after total knee arthroplasty. **a** Image of the length of occlusion. **b** After recanalization, a contained rupture is shown as contrast leakage toward the implanted prosthesis. **c** Postinterventional result with restored vascular continuity after stentgraft implantation

Eleven patients underwent primary endovascular treatment, of which ten procedures were performed successfully: (stent) PTA (*n* = 6), aspiration thrombectomy (*n* = 5), thrombin injection (*n* = 1), and embolization (*n* = 1); combinations of procedures are presented in Fig. 4. In one case, the bleeding was no longer detectable in the DSA, whereas no intervention was necessary*.* The endovascular approach failed in one case, which led to surgical conversion.

**Fig. 5:**
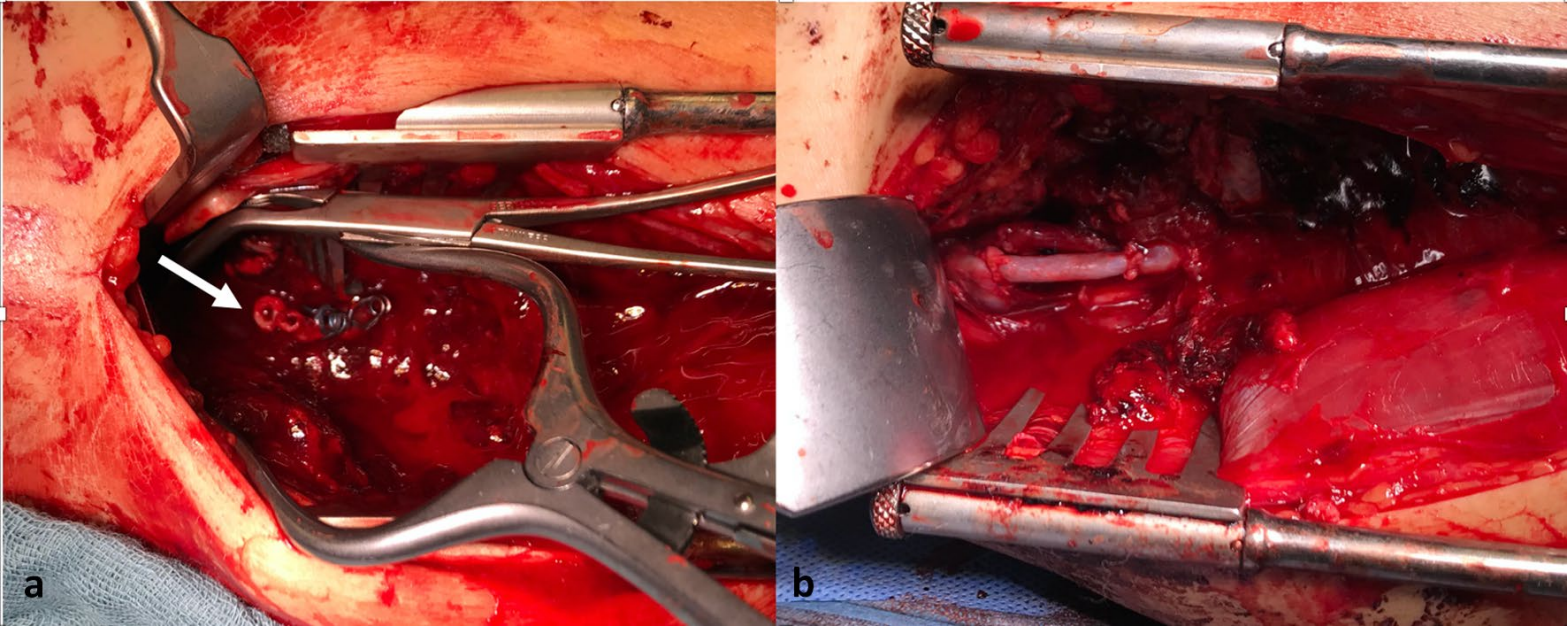
Intraoperative findings with sharp transection of the popliteal artery and vein (**a**, white arrow), and after reconstruction with autologous vein interposition (**b**)

A total of six patients underwent surgical treatment. The procedures performed included bypass/graft interposition in four cases, one patch plasty and one hybrid approach, respectively. The used graft material was autologous vein exclusively (Fig. 5). The hybrid procedure consisted of an endovascular stent graft implantation, followed by fasciotomy of the lower leg. Average blood loss during surgery was 550 ml (200–1200 ml).

Table 3 provides an overview of the results of the reconstructive vascular therapy, divided into surgical and interventional procedures. There was a significant difference between the two groups in the duration of the respective procedures with a shorter time required for endovascular therapy. There were no differences with regard to anticoagulation at discharge.

**Table 3:** Vascular procedure

	Total number *n* = 16	Endovascular *n* = 10	Surgery *n* = 6	*P* value
Procedure time (M ± SD)	154.87 ± 116.57	98.89 ± 63.52	238.50 ± 131.26	0.01
Age (y)	67.50	72.00	53.50	0.09
BMI > = 30	7	5	2	0.63
Limb preservation	16	10	6	–
Functional impairment	6	2	4	0.12
Fasciotomy	7	2	5	0.03
LOS (d) M ± SD	16.00 ± 12.68	14.10 ± 14.08	19.17 ± 10.30	0.31
LOS on ICU (M ± SD)	0.50 ± 1.75	0.10 ± 0.32	1.17 ± 2.86	0.79
Reinterventions during follow-up	3	2	1	0.52
Anticoagulant drugs at Discharge				
None ASS DAPT 4 to 8 weeks DAPT permanent Anticoagulation	3 7 3 1 2	2 4 3 0 1	1 3 0 1 1	1.00 1.00 0.25 0.38 1.00

*M* mean, *SD* standard deviation, *BMI* body mass index, *LOS* length of stay, *ICU* intensive care unit, *ASS* acetylsalicylic acid, *DAPT* dual anti-platelet therapy

All treated extremities could be preserved. Functional impairment remained in six cases, half of which occurred in patients with an ischemia time greater than 10 h or late diagnosis in the subacute interval. Fasciotomy was more commonly performed during open surgery.

Functional limitations included persistent swelling, hypo-/dysesthesias, reduction in range of motion, and chronic wound treatment especially after fasciotomy.

Table 4 provides an overview of the therapeutic outcome in correlation to the time of ischemia. On average, patients were hospitalized for 16 days, and there was no significant difference between surgical or interventional therapy. At discharge, perfusion was documented in 15 cases: patients had either palpable peripheral pulses or in presence of PAD an ankle-brachial index (ABI) above 0.5.

**Table 4:** Outcome correlated to ischemia time

	Total number	Ischemia time < 6 h	Ischemia time > 10 h	Subacute ischemia
	*n* = 16	Endovascular/surgical 6/1	Endovascular/surgical 2/3	Endovascular/surgical 2 / 2
Functional impairment		2/1	0/1	0/2
Need for amputation		0/0	0/0	0/0
Fasciotomy		2/1	0/2	0/2
LOS on ICU (d) (M ± SD)		0.17 ± 0.4/0	0/2.33 ± 4.04	0/0
LOS (M ± SD) (d)		14.00 ± 16.54/19.00	4.50 ± 2.12/2.33 ± 9.29	24.00 ± 5.66/9.50 ± 2.12

*LOS* length of stay, *ICU* intensive care unit, *M*  mean, *SD* standard deviation, *d* days

The median follow-up was 629 days (range 2–2408). Three occlusions of the vascular reconstruction were documented, which led to reintervention: two patients were treated endovascularily, one surgically, respectively. All procedures were successful in terms of restoration of leg perfusion.

## Discussion

To our knowledge, this study represents the largest single-center analysis of iatrogenic popliteal injuries.

In our case series, the high rate of limb preservation and function is striking; in particular, that none of the patients required an amputation stands in stark contrast to previously described amputation rates of up to 42% [6, 7].

Nevertheless, some serious complications and possible re-interventions cause increased morbidity for the patient. There is a risk of long-term consequences, such as swelling, chronic pain, and open wound treatment.

Patient selection criteria can provide preoperative guidance to anticipate an increased risk for vascular injury. A predisposition to intraoperative injury of the popliteal artery exists for patients with older age, increasing ASA status, obesity, and especially in the presence of preexisting PAD, as well as prior orthopedic or vascular surgery in the target area [8–10].

Vascular imaging (e.g., CTA) is recommended preoperatively in the presence of revision surgery or a known anatomic variability to map the course of the vessel in relation to the surgical site. It has been shown to minimize the risk of injury for total hip arthroplasty after vascular imaging [11].

Three injuries in our series occurred in the setting of orthopedic revision procedures or preexisting PAD; in one patient, even both risk factors were present.

A vascular variability is often not known in advance, as it usually does not cause any symptoms. Outflow variations of crural vessels occur in approximately 10% of the total population according to angiographic studies. In less than 2%, there is high branching of the anterior or posterior tibial artery. This means that there are two vessels present at the level of the knee joint gap instead of one. The anatomical variation runs usually closer to the periosteum, exposing it to injury during knee surgery [12, 13]. In the present investigation, we observed two patients with high branching, illustrating the frequency of injury in relation to the regular vascular course.

Palpation and documentation of the foot pulses should be performed preoperatively in every patient. Especially in cases of prediagnosed PAD or diabetes hemodynamics should be evaluated by a vascular physician to assess the severity of macro- or microangiopathy or even media sclerosis. In the event of complications, knowledge of the preoperative blood flow situation supports the assessment of clinical symptoms.

We were unable to obtain information on the preoperative pulse status from any of the available patient files, which may reflect a lack of documentation or a low awareness of the importance of preexisting vascular conditions.

A tourniquet should not be applied in presence of peripheral bypass, because it may lead to graft occlusion [4].

Various recommendations exist to prevent vascular injury during orthopedic surgery. Whether flexion in the knee joint has a protective effect in HTO has not been proven.

In general, it is recommended to use a retractor on the dorsal tibia during sawing, which should be guided close to the periosteum [14].

In an experimental study, it was postulated that the risk of vascular injury was reduced when the saw cut was angulated 10° ventrally [15–17].

Vascular injury may clinically present itself by hemorrhage and/or ischemia, as well as pseudoaneurysm formation [3]. The time to diagnosis can range from emergent/urgent cases to incidental findings, which can have an influence on therapeutic decisions. Basically, a distinction is made between sharp and blunt traumas. Sharp injuries are only noticeable by bleeding when the vessel wall is completely opened. Since the procedures are often performed with tourniquet, the detection of bleeding may be difficult. In addition, in our study, only a few patient files contained mentioning of intraoperative abnormalities.

In case of patient transfer to a vascular surgery department with an applied tourniquet, the duration of ischemia should be recorded and is of meaning for the subsequent vascular treatment.

Post-procedural clinical review of pulse status, motor function, and sensitivity is mandatory following orthopedic surgery. Assessment of sensorimotor function may not be adequately possible when pain catheters or local anesthetic procedures are used.

Thus, palpation of the foot pulses or assessment of the ABI has an important role in the objectification of perfusion. Since TKA usually affects middle-aged or older patients, ABI can be influenced by preexisting PAD or media sclerosis in diabetic patients and result in incorrect measurements and therefore should be compared to the preoperative status. In our study, four patients suffered from PAD.

In the presence of leg ischemia, the reestablishment of blood flow is of importance to prevent functional impairment and should be obtained within 6 h.

This stresses the need for timely availability of vascular surgery expertise, especially in the absence of a vascular department on-site.

Hence, it comes as no surprise that in Germany, the certification as an endoprosthetics center requires mandatory cooperation with a vascular surgeon [18].

Blunt trauma can result from exposure to surgical instruments or extreme patient positioning. Thus, intima injury can cause local thrombus accumulation and lead to ischemia.

Arteriosclerotically affected vessels are particularly at risk due to the loosening of plaque components by local manipulation [4].

If not all vessel wall layers are affected by the trauma, spontaneous healing or formation of pseudoaneurysms may occur. These are encapsulated hemorrhages that often only cause symptoms by compression of surrounding structures, including local nerve damage or venous thrombosis. On average, pseudoaneurysms are only discovered after 15 days in the sonographic workup when deep vein thrombosis or compartment syndrome is suspected. The artery should also be assessed in all cases of the suspected diagnoses mentioned above [19, 20].

In our series, two of the three pseudoaneurysms were also diagnosed only in the subacute interval.

### Algorithm for the orthopedic surgery team

**Fig. 6:**
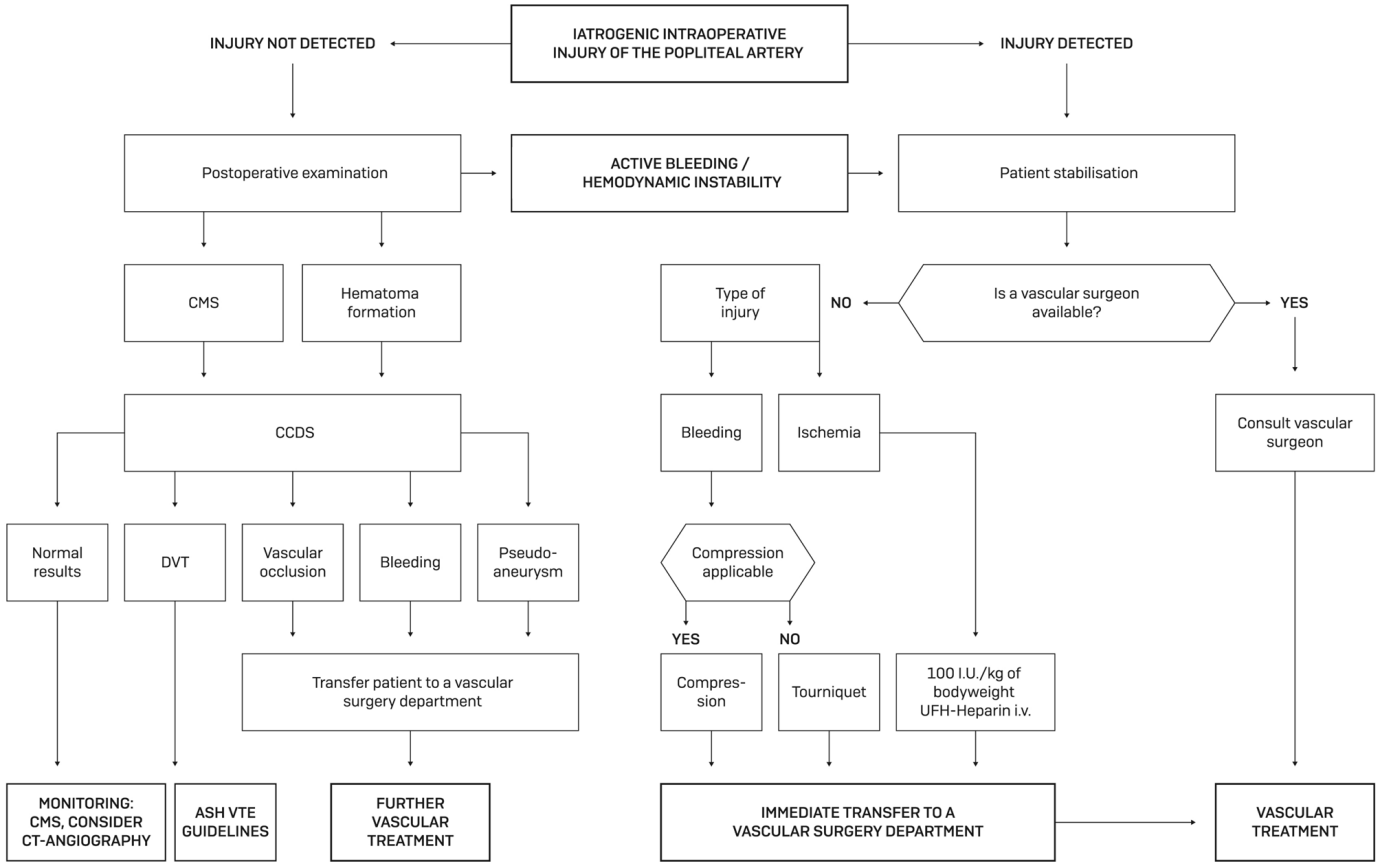
Algorithm for the orthopedic surgery team. *CMS* circulation motion sensation, *CCDS* color-coded duplex sonography, *CT* computed tomography, *ASH* American Society of Hematology, *VTE* venous thrombembolism, *I.U.* international units, *DVT* deep vein thrombosis, *kg* kilograms, *UFH* unfractionated heparin, *i. v.* intravenous)

Based on our interdisciplinary experience in dealing with this complication, we have created an algorithm for postoperative control and examinations to be initiated after completion of the orthopedic procedure and/or in the setting of vascular injury (Fig. 6).

If the injury is noticed intraoperatively or the patient develops hemodynamical instability, basic measures to secure the circulation should be taken first. Close cooperation with the anesthesia department is recommended. If a vascular surgeon is available, he should be called in immediately. Otherwise, rapid contact with the nearest vascular surgery department is necessary.

In case of abnormalities in the postoperative monitoring, such as unusual hematoma formation or CMS abnormalities, CCDS should be performed if available and, depending on the findings, vascular surgery expertise should be obtained.

### Algorithm for vascular physicians

**Fig. 7:**
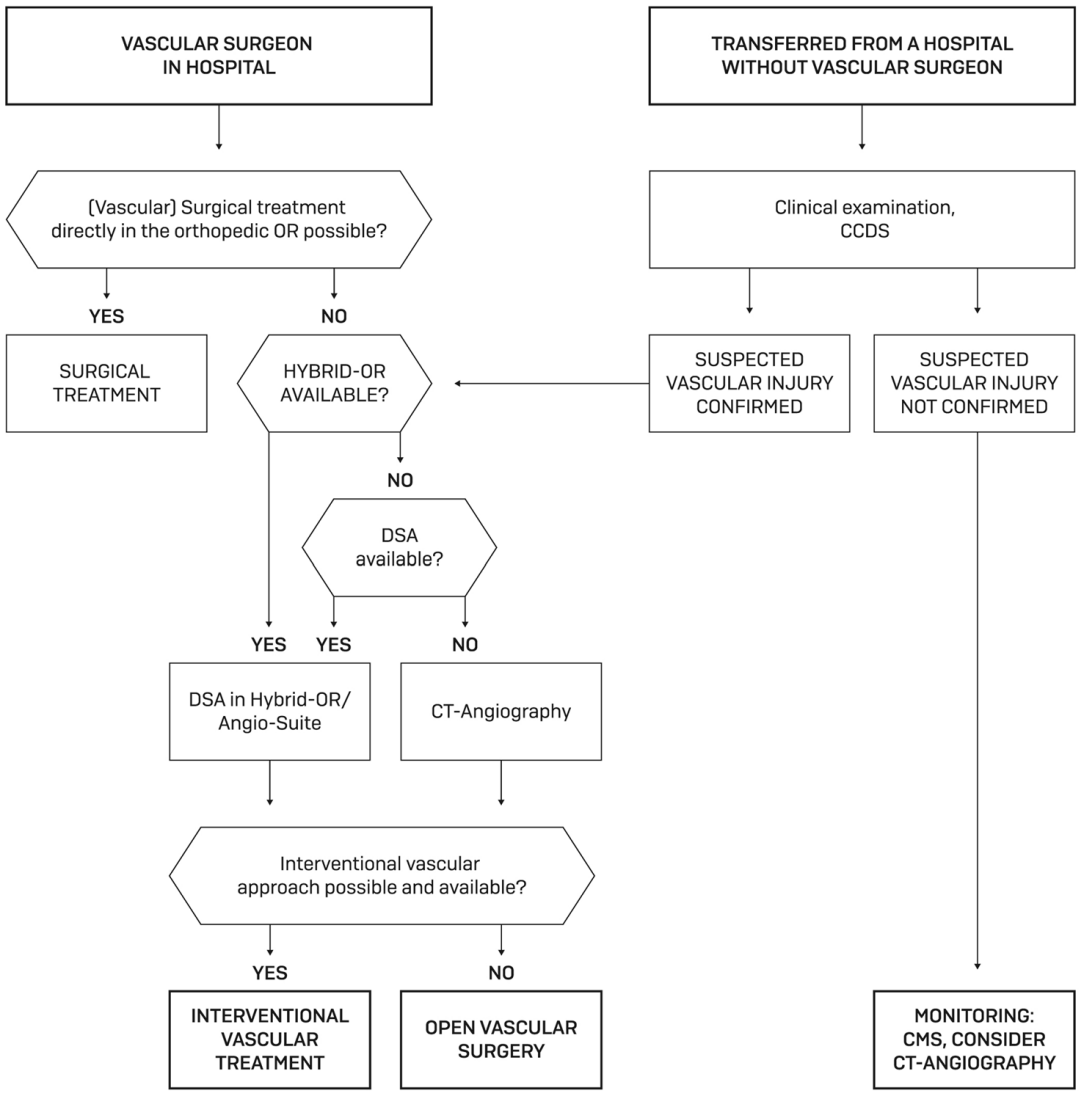
Algorithm for vascular physicians. *DSA* digital subtraction angiography, *CT* computed tomography, *OR* operation room, *TKA* total knee arthroplasty, *CCDS* color-coded duplex sonography, *CMS* circulation motion sensation)

The algorithm for the subsequent vascular treatment is visualized in Fig. 7.

If the suspected diagnosis is clinically confirmed, imaging is necessary to estimate the exact location and type of vascular injury. As described in the results section, the different modalities are used incongruently. We recommend DSA over CT angiography, especially after TKA, because artifacts caused by the inserted prosthesis make it difficult or even impossible to assess the popliteal artery in the knee joint gap. This can be done in an angiosuite or in (hybrid-) OR, depending on site conditions. The choice of therapy is based on clinical assessment of the patient's condition and injury severity. In emergent or urgent cases of hemodynamic instability or already manifest compartment syndrome, surgical therapy may be preferred.

Hybrid surgery was only established during the course of our investigation; therefore, only one case could already be treated accordingly.

### Vascular reconstruction options

Various surgical and interventional procedures are available for vascular reconstruction [21].

The selection of the respective treatment modality should be done interdisciplinary and individually, and depends on the on-site circumstances and availability of vascular expertise, as well as patient condition and severity of the injury.

Intervention has the advantage of less invasiveness and shorter treatment duration [22], which was confirmed by the significantly shorter intervention times in our investigation. In addition, the procedure is viable without general anesthesia. However, due to the patient’s immediate postoperative condition, at least an anesthesia stand-by is recommended. In individual cases, rapidly available endovascular therapy, as stent graft implantation in severe bleeding, can also serve as a bridging measure until definitive surgical repair [23].

Patient age also influences the choice of procedure. In younger patients, stent implantation into the popliteal motion segment should be viewed critically with regard to long-term open rates. In the present study, there was a trend toward endovascular treatment in elderly patients.

Due to the small number of cases, specific data on patency rates after trauma are difficult to obtain. Since also patients with a healthy vascular system are affected by iatrogenic injury, the comparison with PAD patients is of limited value [6]. A case series of three popliteal stent implantations for pseudoaneurysm formation after trauma reported a 100% open rate at 17 months [20].

In cases with thrombus formation caused by an intimal tear or plaque dislocation, an interventional approach with aspiration thrombectomy should be considered. Depending on the size of the lesion and preexisting arteriosclerosis, the additional implantation of a stent (graft) may be necessary.

The length of inpatient stay in our collective was not significantly longer after surgery (19 days) than after intervention (14 days). Compared to usual hospitalization after elective vascular procedures, the length of stay is longer in this collective. This is mainly caused by prolonged reconvalescence after emergency treatment and the longer wound treatment in case of fasciotomy, which makes it difficult to compare to elective procedures.

The development of postischemic compartment syndrome after arterial popliteal injury of various etiologies is common, because it often affects vascularly healthy patients who do not have a preexisting collateral circulation.

The indication for fasciotomy as a preventive treatment should be broad to allow the swelling muscles to expand and thus avoid permanent damage to surrounding structures [2, 24]. If clinical findings are unsure to assess due to the postoperative status and anesthesiologic procedures, a fasciotomy should be performed. In case of manifest compartment syndrome, the procedure is obligatory.

Post-procedural anticoagulation or anti-platelet therapy is necessary after revascularization in most cases. Although no recommendations exist for these rare cases, physicians should consider guidelines for blood thinning medication following PAD treatment as well as individual aspects. For patients with non-arteriosclerotic vessels, a shorter time for medication may be considered. In our institution, we prefer anticoagulation only for bypasses exceeding the knee joint. For stent graft implantation in the popliteal segment, DAPT (dual anti-platelet therapy) is usually administered. Nevertheless, individual factors may oftentimes affect the decision for the medication. An overview on the different therapeutic schemes we used is shown in Table 3.

### Follow-up examinations and re-interventions

Follow-up is important because of the high reintervention rate of 19%. Cause for reintervention was occlusion of the reconstruction in all cases. Two patients were treated endovascularily initially as well as re-intervened. One of those patients suffered from preexisting PAD and developed a re-stenosis. The other patient needed two re-interventions for stent graft clotting. In one case, a bypass occlusion occurred, which was treated by implantation of a new bypass. All cases were successfully re-intervened.

Analogous to other vascular reconstructions, clinical control is recommended at least once a year, to detect and treat stenosis formation at an early stage, for example.

Because these patients often have not had vascular treatments before and are therefore not used to annual examinations, awareness of lifelong follow-up and need of anticoagulant medication is important. In our investigation, eight patients present regularly up to today. During follow-up, two patients died of nonprocedure-associated diseases, and the remaining six discontinued follow-up for unknown reasons.

### Limitations

The main limitation in the validity of the study arises from the small number of cases. In addition, retrospective analysis of the affected patients may not be complete, because a wide variety of ICD-codes was used for documentation. Thus, an unknown number of unreported cases is possible. A prospective trauma registry, as established at our hospital since 2016, offers advantages in this regard. Also, a national registry would add further evidence.

## Conclusions

- Iatrogenic injury to the popliteal artery is a rare but serious complication that can be associated with significant limitations in quality of life and functional impairment or even limb loss for the patient.
- Preoperative assessment and documentation of CMS is mandatory.
- In case of PAD or previous orthopedic or vascular surgery in patient history, the risk of vascular injury is increased.
- The vascular procedures performed are often complex and require special vascular surgery expertise, making the establishment of collaborative models particularly important.
- Due to a relevant reintervention rate, vascular follow-up is recommended regularly.
